# Shotgun metagenomes from productive lakes in an urban region of Sweden

**DOI:** 10.1038/s41597-023-02722-x

**Published:** 2023-11-17

**Authors:** Alejandro Rodríguez-Gijón, Justyna J. Hampel, Jennah Dharamshi, Stefan Bertilsson, Sarahi L. Garcia

**Affiliations:** 1 Department of Ecology, Environment, and Plant Sciences, Science for Life Laboratory, Stockholm University, 10691 Stockholm, Sweden; 2 Department of Aquatic Sciences and Assessment, Swedish University of Agricultural Sciences, 75651 Uppsala, Sweden; 3 Institute for Chemistry and Biology of the Marine Environment (ICBM), University of Oldenburg, 26129 Oldenburg, Germany

**Keywords:** Limnology, Microbial ecology, Environmental impact

## Abstract

Urban lakes provide multiple benefits to society while influencing life quality. Moreover, lakes and their microbiomes are sentinels of anthropogenic impact and can be used for natural resource management and planning. Here, we release original metagenomic data from several well-characterized and anthropogenically impacted eutrophic lakes in the vicinity of Stockholm (Sweden). Our goal was to collect representative microbial community samples and use shotgun sequencing to provide a broad view on microbial diversity of productive urban lakes. Our dataset has an emphasis on Lake Mälaren as a major drinking water reservoir under anthropogenic impact. This dataset includes short-read sequence data and metagenome assemblies from each of 17 samples collected from eutrophic lakes near the greater Stockholm area. We used genome-resolved metagenomics and obtained 2378 metagenome assembled genomes that de-replicated into 514 species representative genomes. This dataset adds new datapoints to previously sequenced lakes and it includes the first sequenced set of metagenomes from Lake Mälaren. Our dataset serves as a baseline for future monitoring of drinking water reservoirs and urban lakes.

## Background & Summary

Healthy lakes and shorelines provide multiple societal benefits and contribute positively to our quality of life and livelihoods. Lakes can be used as sources of drinking water for surrounding urban areas and can also supply water for industry and agricultural irrigation. Lakes also offer ample opportunities for recreation and tourism. However, urbanization of surrounding areas, causing eutrophication and other types of anthropogenic impacts, can pose major threats to the sustainable use of these natural ecosystems. In this way, lakes are not only valuable resources, but also sentinels of anthropogenic impacts and environmental change, as their microbiomes are highly sensitive to perturbations, and respond rapidly and predictably to changing environmental conditions^1–4^. In depth and high-quality records of the current state of lake microbiomes can thus be used as a baseline to assess change and anthropogenic impacts on lake water quality. However, we face a paucity of such metagenomic data that could provide us with more deep and insightful information about microbial diversity and the genome-encoded functional traits of such communities. Here, we release metagenomic data (Table 1 and Table S1) and metagenome-assembled genomes (MAGs)^5^ from several urban and anthropogenically impacted Swedish lakes. Most of these lakes have previously been studied and characterized in terms of limnological features and water chemistry, but information on their microbial communities is scarce.

**Table 1:** Sampling locations, dates of sample collection, extraction method, and number of SRGs per sample.

Sample name	Lake	Date	Extraction method	Number of SRGs found in the sample
Sample_104_S78	Ekoln	Aug-2002	Qiagen	57
Sample_105_S79	Erken	Aug-2002	Qiagen	52
Sample_101_S75	Limmaren	Aug-2002	Qiagen	93
Sample_103_S77	Norrviken	Aug-2002	Qiagen	51
Sample_102_S76	Valentunasjön	Aug-2002	Qiagen	103
Sample_107_S7	Mälaren_B	05-Aug-2021	MP Bio	83
Sample_104_S4	Mälaren_D	05-Aug-2021	MP Bio	84
Sample_102_S2	Mälaren_B	11-Aug-2021	MP Bio	71
Sample_113_S84	Mälaren_B	21-Jul-2021	Qiagen	52
Sample_108_S8	Mälaren_B	21-Jul-2021	MP Bio	62
Sample_103_S3	Mälaren_D	21-Jul-2021	MP Bio	51
Sample_110_S81	Mälaren_B	24-Aug-2021	Qiagen	57
Sample_109_S9	Mälaren_B	24-Aug-2021	MP Bio	78
Sample_111_S82	Mälaren_D	24-Aug-2021	Qiagen	67
Sample_105_S5	Mälaren_D	24-Aug-2021	MP Bio	76
Sample_107_S80	Trehörningen	30-Aug-2021	Qiagen	73
Sample_106_S6	Långsjön	30-Aug-2021	MP Bio	58

For more metadata including latitude, longitude, depth, and temperature see Table S1.

**Fig. 1:**
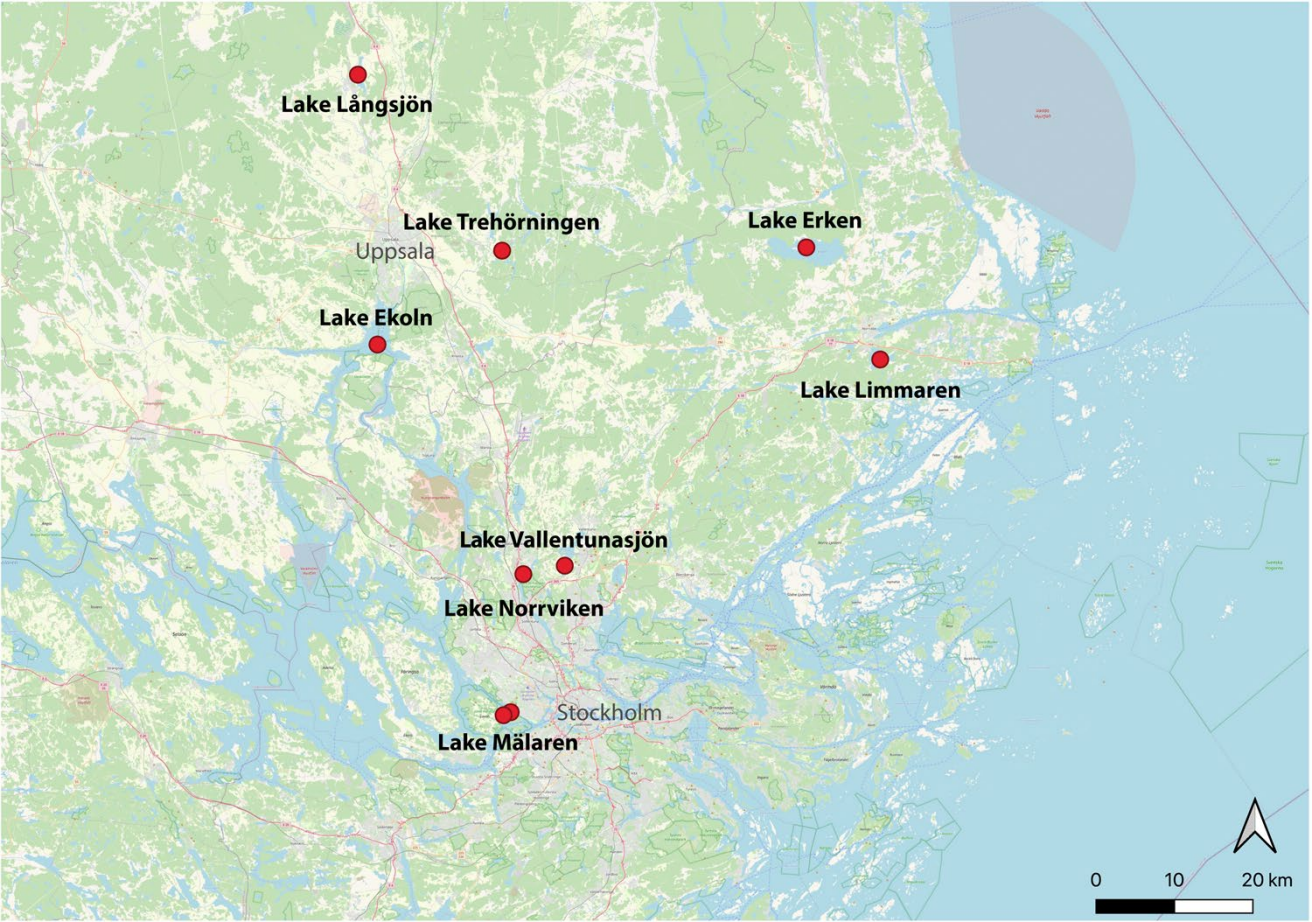
Map of sampling locations. Note that Mälaren was sampled at two nearby but different locations (named Mälaren_B and Mälaren_D in supplementary data files). For coordinates and metadata, see Table S1.

Lake Mälaren is the third largest lake in Sweden, and according to the Mälaren Water Protection Association (Mälarens vattenvårdsförbund), it serves as the main drinking water supply to approximately 2 million residents in Sweden. The lake receives high nutrient loads from surrounding agricultural areas and has a history of recurrent cyanobacterial blooms^6^. Moreover, the eastern part of the lake drains into the Baltic Sea, transporting nutrient rich waters into the vulnerable coastal zones^7^. Despite its significance, Lake Mälaren is severely understudied and the microbial community composition in the lake has only been superficially characterized. This comprehensive metagenomic dataset is thus the first detailed insight into the bacterial dynamics of Lake Mälaren, and was obtained during the 2021 summer season. For comparison, we sampled two productive lakes (Trehörningen and Långsjön; Fig. 1) in the vicinity of Uppsala to contrast and compare variation in microbial communities both within and between similar lakes from the same region of Sweden.

Additionally, we sequenced and assembled metagenomes from five previously sampled eutrophic lakes in the urban Stockholm-Uppsala region: lakes Ekoln, Erken, Limmaren, Vallentunasjön, and Norrviken. These samples, collected in 2002, have previously been characterized for their bacterial composition using less comprehensive and now outdated methods (i.e., clone libraries and sanger sequencing, and terminal-restriction fragment length polymorphism, T-RFLP)^8,9^. These five highly eutrophic lakes have a long history of seasonal cyanobacterial blooms in the summer^8^ and feature pronounced seasonal dynamics within the bacterioplankton communities^9,10^. In brief, Lake Ekoln is a subbasin in the northern part of Lake Mälaren. It stratifies in summer and receives high nutrient inputs from the city of Uppsala and the surrounding agricultural areas. Lake Erken, located east of Uppsala, is a eutrophic lake that is thermally stratified during the summer and has been extensively studied for several decades^11^, also with regards to microbial community composition^12–14^. Lake Erken also serves as a backup drinking water reservoir for the nearby city of Norrtälje. Lake Limmaren, located 70 km north of Stockholm, receives high nutrient loads from sediments and has a long history of dense blooms of *Microcystis*, *Anabaena*, and *Aphanizomenon*^8^. Historical accumulation of nutrients from urban settlements also plays a significant role in the state of the hypereutrophic lake Vallentunasjön located in a suburban area north of Stockholm^10^. The lake has undergone major restoration efforts, but still suffers from eutrophication with frequent cyanobacterial blooms. Lastly, lake Norrviken in Stockholm has received high historical loads of domestic and industrial sewage in the past and is also subjected to intense cyanobacterial blooms^15^.

**Fig. 2:**
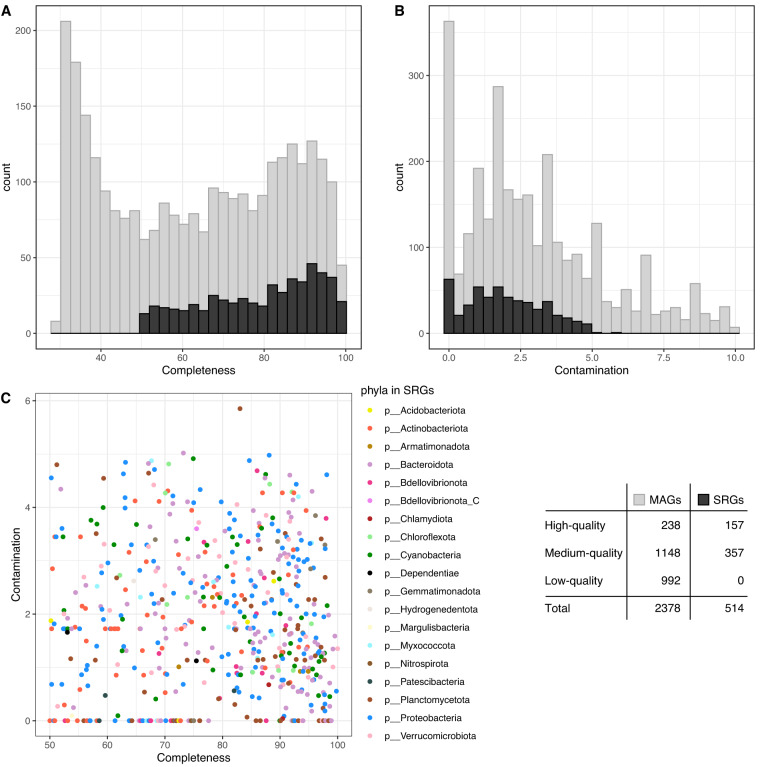
Quality of the MAGs. Completeness (**A**) and contamination (**B**) across all the 2378 metagenome-assembled genomes (MAGs, in grey). Highlighted in black, the 514 species representative genomes (SRGs). Correlation between completeness and contamination for all 514 SRGs, colored by phyla (**C**). The legend table indicates the number of MAGs and SRGs classified as high-quality (>90% completeness and <5% contamination), medium-quality (≥50% completeness and <10% contamination), and low-quality (<50% completeness and <10% contamination), following the MIMAG standards for MAG quality completeness and contamination cutoffs^35^.

**Fig. 3:**
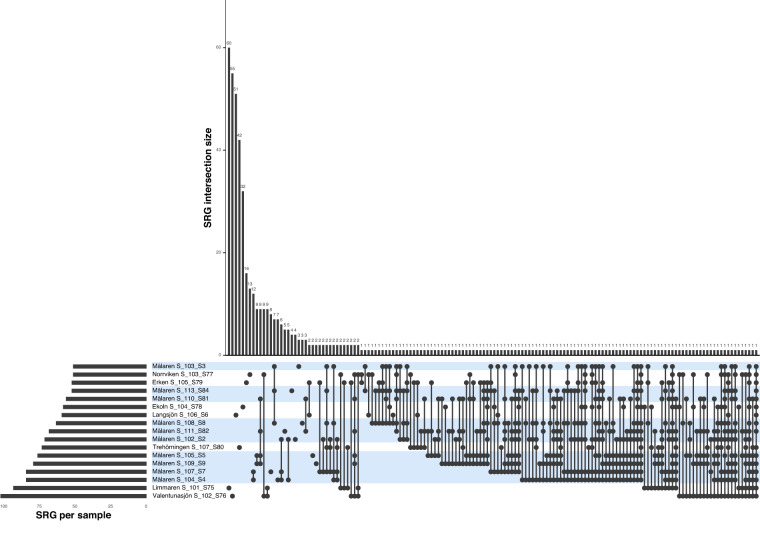
Intersection plot of shared species representative genomes (SRGs) across samples. Intersection size shows the number of SRGs present in each sample. The sum of all intersection sizes equals the 514 SRGs in the dataset. For example, we find that 60 SRGs are exclusively present in Limmaren Sample 101 S75. Set size is the number of SRGs present in total per sample. Samples highlighted in blue are from Lake Mälaren.

**Fig. 4:**
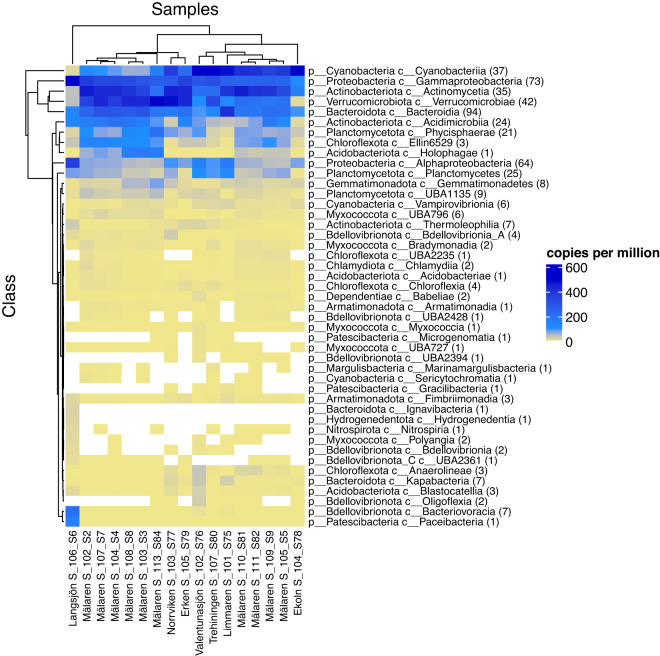
Heatmap indicating the sum of genome copies per million reads per class for each bacterial class represented by the SRGs per sample. White indicates that reads from the given class were not detected in the sample. In the figure, the color yellow/khaki starts at 0.0001 genome copies per million reads. The heatmap is hierarchically clustered.

Our broader ambition was to collect and sequence data that could be used to provide a comprehensive view of microbial communities in urban lakes of the greater Stockholm area, with special emphasis on Lake Mälaren (Table S1). Such data could also be used to identify microbial health hazards (such as pathogens), serving as a baseline for future monitoring efforts based on microbiomes as sentinels of environmental health. Finally, this dataset could be used to generate novel hypotheses on linkages between lake microbiomes and human activities in the watershed. We thus release 17 shotgun metagenomes (Table S2) and their corresponding single-sample assemblies. In addition, we performed genome-resolved metagenomics and obtained 2378 MAGs (>30% completeness and <10% contamination) (Fig. 2 and Table S3). We then clustered MAGs from across all samples together based on 95% average nucleotide identity (ANI) and obtained 514 species representative genomes (SRGs; >50% completeness and <6% contamination) (Fig. 2). We also provide an overview of the number of SRGs specific to and shared between the different sampled lakes (Fig. 3) and relative abundance patterns of different classes of Bacteria represented by SRGs across the lake metagenomes (Fig. 4 and Table S4).

## Methods

### Sampling

Surface water samples from Swedish lakes were collected in both 2002^8^ and 2021 (Fig. 1). Samples from lakes Ekoln, Erken, Limmaren, Norrviken, and Vallentunasjön were collected in August 2002 and their bacterial 16 S rRNA gene composition was previously superficially described using molecular cloning and sanger sequencing^8^. We retrieved one membrane filter (Supor, Gelman) from each of those lakes from a −80 °C freezer, where they have been stored since 2002. Samples from lakes Mälaren (2 locations in Stockholm: Drottningholm - D and Brostugan - B), Trehörningen (Uppsala), and Långsjön (Uppsala) were collected in July and August 2021 (Table 1). In these cases, surface water was collected from a wooden deck with a Limnos tube-sampler (Limnos, Poland) and 300 mL hand-filtered in duplicate onto 0.2 µm Sterivex filters (Millipore) that were subsequently frozen at −20 °C until DNA extraction. Environmental parameters (temperature, dissolved oxygen, and conductivity) were measured using a YSI sonde (Table S1).

### DNA extractions

For all samples, DNA was extracted using the DNAeasy PowerWater kit (Qiagen) following the manufacturer’s instructions and DNA concentrations were measured using a Qubit dsDNA HS kit (Thermo Fisher Scientific Inc). However, for some of the samples the Qiagen kit did not yield high quality DNA. Additionally, the duplicate filters from 2021 were selected for extractions at Linneaus University using the FastDNA® SPIN Kit for soil (MP Biomedicals) with a modified cell lysis step to ensure extraction of cyanobacteria. First, 1467 µL of Sodium Phosphate Buffer (from kit), 183 µl MT buffer (from kit), and 16.5 µl of Proteinase K solution (MP Biomedicals) were added to the Sterivex filters. The filters were mixed, tightly capped, wrapped in parafilm and incubated overnight (~15 hours) in a rotating oven at 55 °C. This was done to ensure extraction of DNA from low biomass samples and to improve cell lysis. Following the overnight incubations, samples were extracted following the manufacturer’s protocol. All DNA concentrations were measured using the Qubit dsDNA HS kit (Thermo Fisher Scientific Inc). All samples that yielded DNA were sent for sequencing.

### Library preparation and sequencing

Sequence libraries were prepared using SMARTer Thruplex library preparation (350 bp average fragment size) at the National Genomics Infrastructure (NGI) at the Science for Life Laboratory (SciLifeLab) in Stockholm. Sequencing was done on the Illumina NovaSeq 6000 platform using a S4 v1.5 flowcell in 300 cycle mode (2 × 150 bp). The Bcl to FastQ conversion was performed using bcl2fastq_v2.20.0.422 from the CASAVA software suite at NGI. Sequences were demultiplexed and quality control and raw data were retrieved on an HPC server hosted by the Swedish National Infrastructure for Computing (SNIC).

### Analysis of raw sequence reads

Processing of raw sequence reads was performed using the metaWRAP pipeline^16^ (v1.3.2). Forward and reverse reads were first trimmed using the “read_qc” module with default settings and TrimGalore^17^ (v0.5.0). Final trimmed reads were assembled into metagenome assemblies using the “metaWRAP_assembly” module with MegaHit^18^ (v1.1.3). Short scaffolds (<1000 bp) were discarded by default before assessing assembly quality statistics using QUAST^19^ (v. 5.0.2) (Table S2). Assembly of contigs were subsequently binned into MAGs using the “metaWRAP_binning” module. Briefly, this module performs binning using three metagenomic binning tools: CONCOCT^20^ (v1.0), metaBAT2^21^ (v2.12.1) and maxBIN2^22^ (v2.2.6). Bins generated from these three tools were then consolidated and refined using the “metaWRAP_bin_refinement” module with cutoffs of above 30% for completeness and below 10% for contamination, resulting in MAGs (Table S3). The final bin set was assessed by CheckM^23^ (v1.1.3) for completeness, contamination, and other statistics. All bins with quality completeness >30% and contamination <10% were considered as MAGs (for detailed statistics see Fig. 2) and included in further analyses.

Taxonomic classification of MAGs was performed using GTDB-tk^24^ (v1.5.0) according to GTDB classification^25^ (data release version r202). Taxonomy of all 2378 MAGs can be found in Table S3. All MAGs were then dereplicated at the species-level using dRep^26^ (v3.0.0) with default settings, which resulted in 514 genomes that represent the species present across all of the lakes (SRGs; Fig. 3). In this pipeline, MAGs were first compared with a rapid primary algorithm MASH^27^ and then a secondary clustering algorithm ANIm was run based on an Average Nucleotide Identity (ANI) threshold of 95%, genome completeness of > = 50%, and contamination < = 5%. The most complete and least contaminated MAGs were selected as species representatives (Table S3). Finally, the “metaWRAP_quant_bin” module was used to estimate the relative abundance of SRGs across all samples. The “metaWRAP_quant_bins” module estimates the abundance of MAGs across the sampling using Salmon^28^ (v1.9.0) to index the metagenomic assembly and align reads from each sample to the assembly. Coverage tables were generated estimating the abundance of each contig in each sample in genome copies per million reads (Fig. 4 and Table S4).

To estimate the total number of reads mapped per metagenome, we mapped all 514 SRGs to all trimmed clean reads using Bowtie2 (v2.5.1)^29^. An index was created using the function *bowtie2-build* calling all 514 SRGs, and then mapped against all 17 metagenomes using default parameters. The resulting sam files were converted into bam files, and then used to count the number of mapped clean reads using SAMtools^30^ (v1.17). These results are reported in Table S1.

A map of the sampling locations was constructed in ArcGIS (v3.28 Firenze). Figures depicting SRG completeness and contamination were generated using ggplot2 (v3.3.5) in RStudio (v2022.02.3 + 492). The intersection graph of shared SRGs across samples was generated using the UpsetR package^31^ (v1.4.0). To obtain the total number of genome copies per million reads for every represented bacterial class (Fig. 4), we took the genome copies per million for each SRG (Table S4) and summed them per bacterial class. We then calculated the number of SRGs per class using the function *percencat* from the package plada (v0.1.0; https://github.com/alejandrorgijon/plada_package). The heatmap was generated using the R package ComplexHeatmap^32^ (v2.10.0) and hierarchical clustering was performed with default settings.

## Data Records

All raw read sequence files and single-sample metagenome assemblies are available at the European Nucleotide Archive (ENA) under the BioProject accession PRJEB54817^33^. All 2378 MAGs have been deposited in a SciLifeLab Figshare data repository^5^: 10.17044/scilifelab.22270225.v3. The 514 SRGs have also been deposited under NCBI Bioproject PRJNA1021391^34^. Statistics for raw reads, assemblies, refined high-quality bins, and dereplicated MAGs (SRGs) are provided in the supplementary tables, and include MAG and SRG IDs and taxonomy, MAG membership in SRGs, presence and relative abundance estimates of SRGs across samples, and genome information (Tables S1–S4).

## Technical Validation

The quality of the raw reads was monitored and certified by the National Genomics Infrastructure (NGI) in Solna, Sweden according to accreditation by Swedac ISO/IEC 17025. The quality scale used is Sanger/phred33/Illumina 1.8+. Quality distribution showed Q30 aggregated percentage of bases to be higher than 89 for all metagenomes. PHRED score was 36 for all samples (Table S1). The quality of the MAGs that compose the SRGs was computed with CheckM^23^ (v1.1.3).

### Supplementary information

Table S1

Table S2

Table S3

Table S4

## Data Availability

No custom code was used in this project.
